# Congenital Middle Mesocolic Hernia: A Rare Cause of Neonatal Intestinal Obstruction

**DOI:** 10.21699/jns.v5i4.371

**Published:** 2016-10-10

**Authors:** Elias Chamely, Brice Antao

**Affiliations:** Department of Paediatric Surgery, Our Lady's Children's Hospital, Crumlin, Dublin 12, Republic of Ireland

**Keywords:** Congenital, Middle mesocolic hernia, Neonatal intestinal obstruction

## Abstract

Congenital mesocolic hernia is an extremely rare, but serious cause of intestinal obstruction in children. Given the rarity of this condition, delays in diagnosis and management can have catastrophic consequences. Congenital mesocolic hernias are usually caused by an abnormal rotation of primitive mid-gut and are divided into left and right congenital mesocolic hernias. We report and discuss the clinical and radiological features and management of a neonate with an extremely rare variant, congenital middle mesocolic hernia along with a literature review of this rare condition.

## CASE REPORT

A preterm male neonate of 34 weeks gestation having antenatal scans showing dilated loops of bowel and an enlarged stomach, prompting early investigations soon after birth. He was stable at birth and his clinical examination was unremarkable, apart from bilious nasogastric aspirates. Abdominal plain film showed dilated bowel loops in the upper abdomen with paucity of gas in the rest of the abdomen (Fig.1). An upper gastrointestinal (UGI) contrast study (Fig.1), demonstrated free flow of contrast into a markedly distended duodenum with normal positioned duodeno-jejunal (DJ) flexure to the left side of the midline. There was a significant hold up of contrast at the DJ flexure, with a small amount of contrast trickling into a normal calibre jejunum. These findings were suggestive of a partial obstruction of the duodenum at the DJ flexure.

**Figure:**
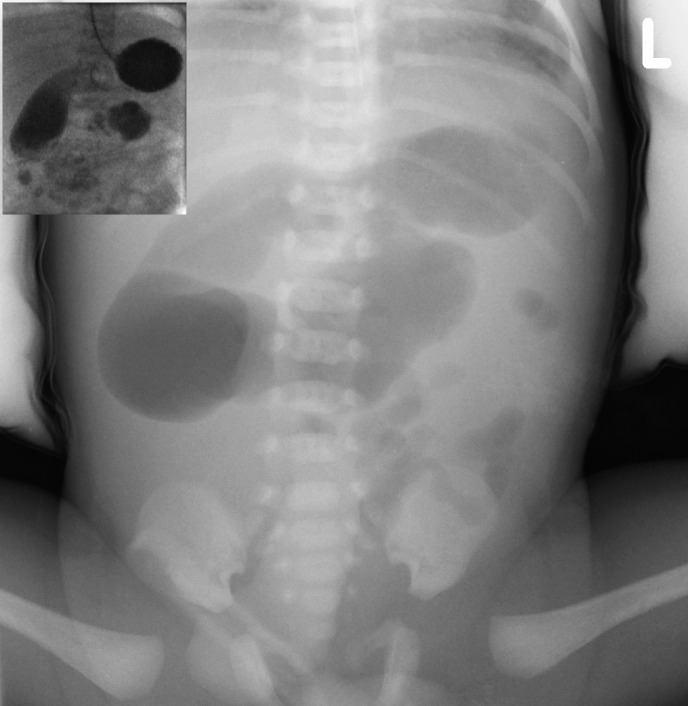
Figure 1: Plain abdominal film showing a single dilated bowel loop in the centre of the abdomen immediately inferior to the body of the stomach. Inset shows UGI contrast study that demonstrated the contrast flowing freely into a markedly distended duodenum as far as the DJ flexure, which appeared in the normal position. A small amount of contrast entered a normal calibre jejunum.

At laparotomy, on the 2nd day of life, the caecum was found to be mobile and positioned high in the right side of the abdomen. There was no evidence of malrotation and the DJ flexure was normally positioned to the left of the midline. However, there was gross dilatation of the duodenum up to the DJ flexure, with the duodeno-jejunal loop of bowel herniating through a small defect in the transverse mesocolon to the left of the middle colic artery causing extrinsic compression at this level (Fig.2,3). The duodenum was kocherised and the herniated loops were reduced and the hernial defect in the transverse mesocolon closed with absorbable sutures. As the DJ flexure was now in an abnormal position out of the hernial defect, and in view of the high mobile caecum, a Ladd's procedure was performed. His post-operative course was uneventful, and he was slowly established to full enteral feeds and discharged home three weeks post-surgery. He continues to thrive well at one year of age with no concerns.

**Figure:**
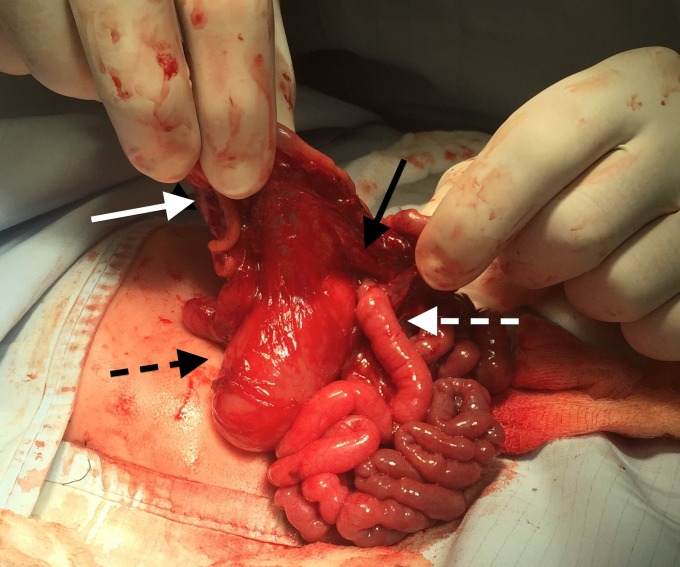
Figure 2: Intraoperative picture which demonstrates a mobile caecum which was near the liver (solid white arrow), and a dilated duodenum (dashed black arrow) and D-J flexure herniating through a defect in the transverse mesocolon (solid black arrow), with a normal calibre jejunum (dashed white arrow) distally.

**Figure:**
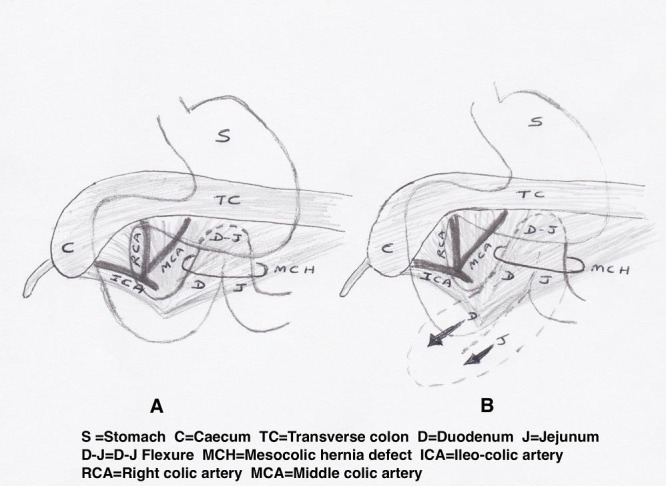
Figure 3: Diagrammatic representation of intra-operative findings of (A) a defect in the transverse mesocolon left of the middle colic artery with the D-J flexure herniated through this defect (B) Arrow heads showing the reduction of D-J flexure, Duodenum and Jejunum out of the hernia defect resulting in the D-J flexure being in a malrotated position.

## DISCUSSION

Mesocolic hernias have incidence between 0.2% and 0.9%, and they constitute up to 5.8% of all cases of small bowel obstruction [1,2]. Congenital mesocolic hernias make up only 5%-10% of all internal hernias and are usually caused by an abnormal rotation of primitive mid-gut with 75% of mesocolic hernias occurring on the left and 25% on the right [3-5]. The left mesocolic hernia originates lateral to the fourth part of the duodenum, behind the inferior mesenteric vein and ascending left colic artery. The right mesocolic hernia protrudes into the ascending mesocolon behind the superior mesenteric artery and inferior to the third portion of the duodenum. In congenital transverse mesocolic hernias, the transverse colon is invaginated behind the superior mesenteric artery and the mesentery, with the duodenum lying anterior to the superior mesenteric artery. However, congenital middle mesocolic hernias protrude into a congenital defect in the middle portion of the transverse mesocolon to the left of the middle colic artery is extremely rare, as was seen in our case [6].There are 9 reported cases of congenital mesocolic hernias in the literature, and only one of them was a neonate who in fact was found to have right mesocolic hernia [5-10].

The clinical features in relation to a congenital middle mesocolic hernia can be quite dramatic compared to the more common left and right-sided mesocolic hernias. This is because the bowel herniates through a very small rent in the mesentery with no sac, causing a tight ring of compression at this point. Also as these cases have a normal rotated gut and fixed D-J flexure, there is limited movement of the bowel within the defect leading to strangulation and incarceration, as seen in the adult case [10]. This was also the mechanism for complete transection of the small intestine due to blunt abdominal injury in the 14-year-old child [6]. In our case, prompt surgical intervention, was based on antenatal suspicion of intestinal obstruction, and inconclusive UGI contrast study in a neonate with bilious vomiting. Although UGI contrast study ruled out malrotation, the level of obstruction at the D-J flexure was more in keeping with an extrinsic compression rather than an intrinsic cause such as duodenal web or stenosis. Also, unlike extrinsic compression with Ladd's bands, as seen in malrotation, the obstruction in this case was due to a normally placed D-J flexure being obstructed as a result of herniation through a narrow middle mesocolic hernia defect. Delay in the diagnosis or surgical intervention, could have led to strangulation and incarceration of the herniated bowel through the small hernia defect. The bowel dilatation on antenatal scan and early post-natal presentation, with a gross dilatation of the duodenum, suggests a prenatal herniation, similar to the other reported case in a term neonate [5]. Similar to malrotation, prompt surgical intervention is advocated, especially when the findings on UGI contrast study is inconclusive and not typical of the commonly considered differential diagnosis of neonatal intestinal obstruction. It is important to note that, absence of malrotation does not rule out a CMH, as this is not associated with its rare variant, congenital middle mesocolic hernia.

## Footnotes

**Source of Support:** Nil

**Conflict of Interest:** Nil
